# Evaluation of Nailfold Capillaroscopy as a Novel Tool in the Assessment of Eosinophilic Granulomatosis with Polyangiitis

**DOI:** 10.3390/jcm14155311

**Published:** 2025-07-28

**Authors:** Gianluca Screm, Ilaria Gandin, Lucrezia Mondini, Rossella Cifaldi, Paola Confalonieri, Chiara Bozzi, Francesco Salton, Giulia Bandini, Giorgio Monteleone, Michael Hughes, Paolo Cameli, Marileda Novello, Rossana Della Porta, Geri Pietro, Marco Confalonieri, Barbara Ruaro

**Affiliations:** 1 Pulmonology Unit, Department of Medical Surgical and Health Sciences, Hospital of Cattinara, University of Trieste, 34149 Trieste, Italy; 2 Biostatistics Unit, Department of Medical Sciences, University of Trieste, 34149 Trieste, Italy; 3 Department of Experimental and Clinical Medicine, Division of Internal Medicine, Azienda Ospedaliero Universitaria Careggi, University of Florence, 50134 Florence, Italy; 4 Department of Cardiovascular and Pulmonary Sciences, Catholic University of Sacred Heart, 00168 Rome, Italy; 5 Division of Musculoskeletal and Dermatological Sciences, Faculty of Biology, Medicine and Health, The University of Manchester Salford Royal NHS Foundation Trust, Manchester M6 8HD, UK; 6 Respiratory Diseases Unit, Department of Medicine, Surgery and Neurosciences, University of Siena, 53100 Siena, Italy; 7 Pulmonology Unit, Ospedale di Gorizia e Mondalcone–SC Patologie Respiratorie, 34170 Gorizia, Italy

**Keywords:** EGPA, ANCA-associated vasculitis, nailfold video capillaroscopy, microvascular abnormality, autoimmune disease

## Abstract

**Background:** Antineutrophil cytoplasmic antibody (ANCA)-associated vasculitis (AAV), including granulomatosis with polyangiitis (GPA), microscopic polyangiitis (MPA), and eosinophilic granulomatosis with polyangiitis (EGPA), represent a spectrum of systemic disorders characterized by necrotizing inflammation of small- to medium-sized vessels. Nailfold videocapillaroscopy (NVC) is a validated, non-invasive technique routinely employed in the assessment of microvascular involvement in systemic sclerosis and in the differential diagnosis of Raynaud’s phenomenon; its application in the context of AAV, particularly EGPA, has not been investigated yet. The present study aims to assess the presence and the possible pattern of microcirculatory abnormalities detected by NVC in EGPA patients, and to explore potential correlations between capillaroscopic findings and disease activity status. **Methods:** A total of 29 patients with EGPA (19 women and 10 men), aged between 51 and 73 years, and 29 age- and sex-matched healthy controls were retrospectively enrolled between October 2023 and April 2025, after providing informed consent and meeting the inclusion and exclusion criteria. NVC was conducted in both groups to assess various morphological parameters, and mean capillary density was also calculated. **Results:** This study observed the presence of capillaroscopic alterations in the EGPA group, including decreased capillary density (38%), neoangiogenesis (72%), rolling (100%), pericapillary stippling (66%), and inverted capillary apex (52%). Overall, when comparing healthy controls with EGPA patients, microcirculatory abnormalities were significantly more prevalent in the latter. Specifically, scores for neoangiogenesis, capillary rolling, pericapillary stippling, and inverted capillary apex showed *p*-values < 0.001. **Conclusions:** Our study demonstrates a higher prevalence of four nailfold videocapillaroscopic abnormalities in patients with EGPA compared to healthy controls. However, the identification of these capillaroscopic alterations as specific to EGPA requires further confirmation. Ongoing studies aim to explore the potential role of NVC as a diagnostic marker and to investigate its correlation with the clinical manifestations of EGPA.

## 1. Introduction

### 1.1. EGPA and the Other ANCA-Associated Vasculitis

The first European study on vasculitis, the anti-neutrophil cytoplasmic antibodies (ANCA) standardization project, was made in 1996 [1].

According to the 2013 revised Chapel Hill classification, eosinophilic granulomatosis with polyangiitis (EGPA) is classified as a small-vessel necrotizing vasculitis belonging to the antineutrophil cytoplasm antibody (ANCA)-associated vasculitis (AAV) group, which also includes granulomatosis with polyangiitis (GPA) and microscopic polyangiitis (MPA) [2]. Results of several clinical studies demonstrated that these diseases are characterized by inflammation of small blood vessels, endothelial injury, and tissue damage [2,3,4,5,6]. There is, however, substantial heterogeneity among these disorders, especially related to the presentation and prognosis of EGPA, which is unique among AAVs [4,5,6].

In EGPA, 30–40% of patients are ANCA-positive, and mostly have a perinuclear fluorescence-labelling pattern (P-ANCA) with anti-myeloperoxidase (MPO) specificity. This allows classification into two EGPA immunophenotypes: MPO-ANCA–positive patients show a more vasculitic phenotype with more frequent relapses, whereas ANCA-negative patients more often exhibit cardiac and gastrointestinal involvement with poorer prognosis [2,3,4,5,6].

EGPA is one of the less common vasculitis, with prevalence in the general population ranging from 9.0 to 58.6 cases per million and incidence from 0.6 to 7.5 per million person-years [2,7,8,9]. Incidence is notably higher among asthmatic patients, reaching up to 64.4 cases per million person-years [9,10]. EGPA is marked by eosinophil-rich, necrotizing granulomatous inflammation, frequently involving the respiratory tract.

EGPA is characterized by a distinct progression, beginning with rhinosinusitis and nasal polyposis, followed by asthma and an eosinophilic phase involving blood eosinophilia and eosinophil tissue infiltration. These features help differentiate EGPA from other AAVs, and they often preceding the vasculitic phase by several years [11,12,13]. Once the vasculitis process begins, systemic symptoms appear, often accompanied by organ-associated vasculitis symptoms, like glomerulonephritis, mononeuritis multiplex, and necrotic vascular purpura [13,14,15].

Several clinical classification criteria have been proposed for EGPA. The first, defined by Lanham et al. in 1984, included three elements: asthma, blood eosinophilia > 1500/mm^3^, and evidence of vasculitis involving at least two organs [14]. However, these clinical features are not always identifiable. Additional criteria have been proposed, including the 1990 ACR criteria, the 2022 ACR–EULAR criteria, and the Chapel Hill Consensus definitions. While these systems demonstrate established sensitivity and specificity, they were not developed for diagnostic purposes and should be used only to support a potential diagnosis of EGPA [2,16,17].

Validated tools to assess disease activity and severity include the Birmingham Vasculitis Activity Score (BVAS) and the Five Factor Score (FFS) [18,19], whose results are often comparable. However, no standardized biomarker is currently available to assess disease activity in EGPA patients.

### 1.2. Nailfold VideoCapillaroscopy and Its Potential Role in Vasculitis, Focus on EGPA

Nailfold videocapillaroscopy (NVC) is a non-invasive imaging technique that allows detailed assessment of nailfold microcirculation [20]. The digital videocapillaroscope with 200× being the most common magnification used is considered the gold standard, and provides high-resolution images and enables reliable detection of capillary abnormalities, making it suitable for both clinical and research use [21].

NVC is a safe technique that allows the evaluation of microvascular alterations through qualitative, semiquantitative, and quantitative assessments. Qualitative analysis describes the overall microvascular architecture, including the shape and distribution of capillaries. Semiquantitative evaluation assesses features such as giant capillaries, disorganization of capillary architecture, microhemorrhages, neoangiogenesis, and capillary loss (Table 1). Quantitative assessment measures capillary density, identifies the presence of avascular areas and enlarged capillaries, and evaluates the frequency of each alteration [20,22].

Currently, NVC has a well-established role in the evaluation of Raynaud’s phenomenon (RP) and systemic sclerosis (SSc). The scleroderma pattern is defined by the presence of giant capillaries (i.e., capillaries with an apical diameter ≥ 50 μm), progressive capillary loss leading to the formation of avascular areas, and a general disorganization of the capillary architecture [22].

Recently, NVC has become increasingly important as an asset for other diseases, for example, considering its introduction in the routine clinical practice also for AAV and some non-rheumatic diseases, even though its role in this field is still not well assessed [23,24,25,26,27,28,29]. Furthermore, recent findings have identified distinct phenotypes characterized by NVC, including microangiopathic patterns observed in numerous patients with AAV, which appear to correlate with disease activity [27]. NVC may represent a potentially valuable adjunctive technique, allowing for direct assessment of the affected microvascular structures. Nevertheless, the capillaroscopic findings associated with vasculitis are various and currently lack the specificity required for definitive clinical application [23].

In this context, we aim to assess if capillaroscopy may play a key role in the evaluation and longitudinal monitoring of microvascular alterations, especially regarding its potential diagnostic support through the identification of a specific capillaroscopic pattern.

## 2. Materials and Methods

### 2.1. Study Design

The primary aim of this study is to identify a potential correlation between capillaroscopic alterations and pulmonary function tests (PFTs) in order to establish a potential application of nailfold videocapillaroscopy (NVC) in EGPA patients within clinical practice. The secondary aims of this study are the following:

- To determine the differences in capillaroscopic alterations between pANCA-negative and pANCA-positive EGPA patients;
- To assess the prevalence and severity of capillaroscopic alterations in patients with EGPA compared to a healthy control cohort;
- To study the association between capillaroscopic alterations and EGPA activity status;
- To study the association between capillaroscopic alterations and C-reactive protein levels (CRP).

### 2.2. Population of the Study

We conducted a multicenter cross-sectional case–control study of 29 retrospectively selected patients attending Monfalcone, Gorizia, and Our European Reference Network Pneumological Unit with a potential diagnosis of EGPA based on the two main criteria used for the assessment of EGPA: 2022 American College of Rheumatology/European Alliance of Associations for Rheumatology Classification Criteria for Eosinophilic granulomatosis with polyangiitis [17] and 2012 revised International Chapel Hill Consensus Conference Nomenclature of Vasculitides [2] or diagnosis of EGPA confirmed by histological examination and 29 volunteer healthy subjects similar in sex and age who constituted the control group. The control group consisted of healthy individuals with no documented history of autoimmune diseases, as confirmed by their electronic medical records. Inclusion criteria for EGPA group were the following:

- 18 years old or more;
- Diagnosis of EGPA;
- Signed written informed consent.

Exclusion criteria for both groups were the following:

- Minor age;
- Underlying malignancies;
- Systemic untreated infections;
- Heart failure;
- Severe renal impairment and/or dialysis;
- Unsatisfactory asthma control;
- Sarcoidosis;
- Pulmonary hypertension;
- Presence of hypoxia and comorbidities which could represent a bias for microvascular assessment at NVC such as recurrent trauma to the hands, primary biliary cholangitis, diabetes, severe uncontrolled systemic hypertension, peripheral atherosclerotic diseases, inflammatory idiopathic myopathies, systemic lupus erythematosus, systemic sclerosis (SSc), Sjogren’s syndrome, rheumatoid arthritis and multiple connective tissue diseases (e.g., SSc overlapping with SLE or rheumatoid arthritis).

Written informed consent was obtained from each patient and the control group, and they were enrolled between October 2023 and April 2024.

### 2.3. Study Assessment

All patients initially evaluated at our center, as well as at the Pordenone and Gorizia centers, subsequently underwent capillaroscopy at our outpatient clinic, which has been operational since 2021. Additionally, health controls were also assessed at our outpatient clinic. Nailfold videocapillaroscopy (NVC) was performed by two trained operators who were blinded to the subjects’ health status (healthy or affected by EGPA). The procedure employed a 200× magnification probe integrated with image analysis software (DS Medica Srl Videocap©, VideoCap® 3.0, Cernusco sul Naviglio, Italy). All images were captured using the same instrument to ensure consistency and subsequently reviewed by both operators. Image analysis was conducted through consensus between two senior NVC specialists with 16 and 10 years of experience. In cases of disagreement, a third experienced NVC operator with 20 years of expertise provided an additional opinion to resolve discrepancies. Patients who underwent the examination were carefully instructed not to smoke or drink coffee before the examination and were made to sit in a room at 22–25 °C for at least 15 min, as recommended by good examination practice, as reported below [27,28,29]. Data on the respiratory function tests performed at the time of the capillaroscopy were all performed with the same instrument and reviewed by the same pulmonary specialist, according to ERS/ATS technical standard on interpretive strategies for routine lung function tests [30].

Patients were subjected to a comprehensive panel of investigations conducted in parallel, with the aim of assessing their general clinical condition.

Such assessments include the following:

- Demographic, lifestyle and clinical standard information
- Asthma Control Test (ACT): a patient self-administered questionnaire for identifying those with poorly controlled asthma. The scores range from 5 (poor control of asthma) to 25 (complete control of asthma), with higher scores reflecting greater asthma control. An ACT score >19 indicates well-controlled asthma.
- Anthropometric measurements such as height, weight, body mass index and ambulatory blood pressure monitoring.
- Blood tests such as creatinine and eGFR, white blood cell count, glucose levels, inflammatory markers (C-reactive protein and erythrocyte sedimentation rate), IgE titer, autoimmunity laboratory tests (ANA titer and pattern, ENA titer, ANCA titer and pattern).
- Pulmonary function tests (simple and comprehensive spirometry): forced expiratory volume in 1 s (FEV1), forced vital capacity (FVC), FEV1/FVC ratio, Residual volume (RV), total lung capacity (TLC), diffusing capacity of the lung for carbon monoxide (DLCO) and carbon monoxide transfer coefficient (KCO).
- High-resolution computed tomography (HRCT).

### 2.4. NVC Alterations

Evaluation of capillaroscopic parameters involves a qualitative analysis, assessing the architecture, morphology, and distribution of capillaries, abnormalities of individual capillaries, and a quantitative analysis, including capillary density, avascular areas, measurement of capillary diameters and frequency of each abnormality [22,29]. Definitions of capillaroscopic parameters adapted were the following:

- Capillary density: number of capillaries across one millimeter of the distal row, considered low if <7 capillaries/mm.
- Avascular areas: distance > than 500 μm between two adjacent capillary loops from the distal rows.
- Tortuous capillaries: single or multiple crossovers.
- Crossed capillaries: limbs are crossed upon themselves or with another.
- Angiogenesis: meandering and irregular loops, sometimes with a bushy appearance.
- Microhemorrhages: Hemosiderin deposit, seen as dark mass.
- Ectatic capillary: capillary diameter between 20–50 μm.
- Giant capillary: homogeneously enlarged loops with diameter ≥ 50 μm.
- Slow blood flow: markedly slowed or discontinuous flow inside the capillary at the dynamic evaluation at the time of examination.
- Rolling: disrupted flow of red blood cell; this can be observed as these individual cells flowing sequentially through the capillaries in at least 25% of capillaries evaluated in each field of 1 mm.
- Pericapillary stippling: microscopic pigmented deposits that can be typically observed above the apical portion of the capillary segment.
- Inverted capillary apex: It is the result of an anomaly in capillary morphology, in which the typical U-shaped structure is not preserved, and an inversion of the normal pattern is observed.

### 2.5. Statistical Analysis

Categorical variables were expressed as counts and percentage. Continuous variables were expressed as mean (and relative SD) or median (and relative IQR), as appropriate. Comparison of categorical variables between groups was made by chi-square test or Fisher’s exact test. In the case of continuous variables, *t*-test or Mann–Whitney U-test was used, depending on the validity of assumptions. In the case/control comparison, to evaluate the effect of sex and age as potential confounding factors, any *p*-values equal or lower than 0.05 were considered statistically significant.

## 3. Results

We conducted a multicenter cross-sectional case–control study of 29 retrospectively selected patients attending Monfalcone, Gorizia, and Our European Reference Network Pneumological Unit with a potential diagnosis of EGPA based on the two main criteria used for the assessment of EGPA: 2022 American College of Rheumatology/European Alliance of Associations for Rheumatology Classification Criteria for Eosinophilic granulomatosis with polyangiitis [17] and 2012 revised International Chapel Hill Consensus Conference Nomenclature of Vasculitides [2] or diagnosis of EGPA confirmed by histological examination and 29 volunteer healthy subjects similar in sex and age who constituted the control group. The characteristics of our study population are listed in Table 2.

Overall, three patients (10%) in the study underwent capillaroscopic examination despite not receiving any targeted therapy, owing to their clinically inactive disease status. Notably, these patients demonstrated well-controlled disease, with the asthma control test (ACT) ranging from 19 to 24 in over 95% of cases. Additionally, 15 patients (52%) underwent capillaroscopy while receiving corticosteroid therapy, 11 patients (38%) during treatment with methotrexate or other biologic agents, and 12 out of the 15 patients receiving corticosteroid therapy had a treatment regimen that included combination with a biologic agent, as detailed in Table 3.

The information listed above was obtained through a data search relevant to the article on the G2 system, which is our electronic medical record management system. We will add a brief explanation in the Methods section to clarify this point, using the available records as of October 2023.

### 3.1. Characteristics of Capillaroscopy Alterations in EGPA and in Control Group

**Figure 1:**
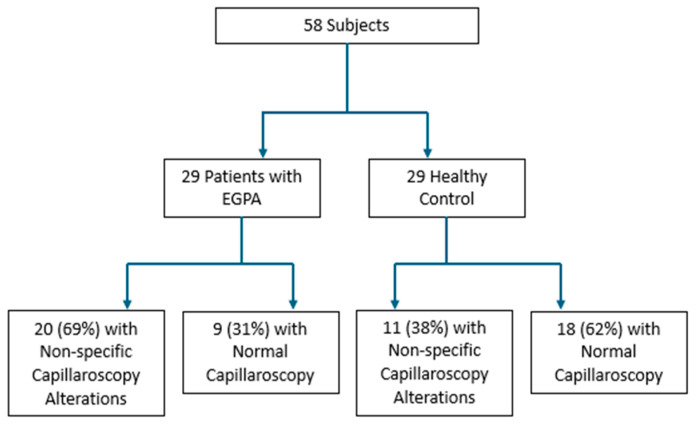
Results of our study on the two groups taken into consideration.

In the group of patients with EGPA, no one had their capillaroscopic examination performed at the time of diagnosis. Study patients were age- and sex-matched to the control group participants, and for both participants NVC was performed by two operators trained in the execution of the method, with a 200× magnifying probe connected with image analysis software (DS Medica Srl Videocap©). Comparison between healthy individuals and patients with EGPA revealed statistically significant microcirculatory abnormalities in the latter group, as illustrated below in Figure 1 and Table 4.

We analyzed the main microcirculatory abnormalities and the frequency with which they occurred in patients with EGPA compared to the control group; the results are displayed in Table 4.

**Figure 2:**
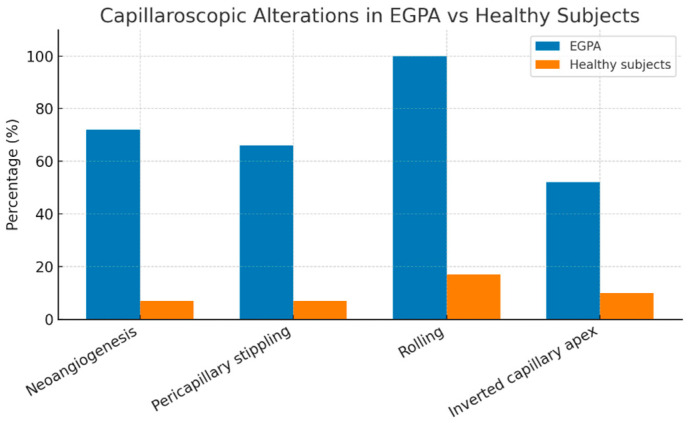
Comparison of the prevalence (%) of capillaroscopy alterations between patients with EGPA and matched healthy subjects. The four alterations (neoangiogenesis, pericapillary stippling, rolling, and inverted capillary apex) are shown. Statistical significance (*p*-value < 0.001) was observed for all these alterations, indicating a higher frequency of microvascular changes in the EGPA cohort.

**Figure 3:**
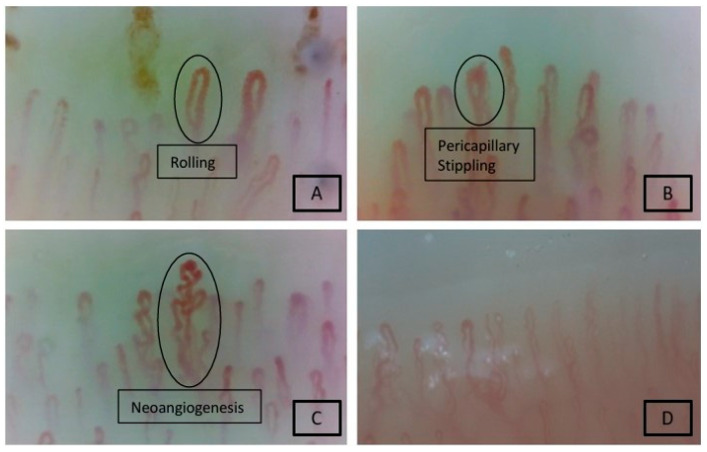
Four patients diagnosed with EGPA: rolling, neoangiogenesis, and the presence of capillary branching (**A**–**D**). Also shown is the characteristic feature of pericapillary stippling (**B**).

We observed statistical significance between the EGPA cohort and the healthy control group concerning these four capillaroscopic alterations: presence of neoangiogenesis, pericapillary stippling, rolling, and inverted capillary apex, indicating that the method was reasonably valid, as reported in Figure 2 and Figure 3, whereas low capillary density did not reach statistical significance.

### 3.2. Relation Between Capillaroscopy Alterations and Pulmonary Function Tests in EGPA Patients

We collected the spirometry parameters of EGPA patients obtained within approximately ±6 months of the capillaroscopy examination. Correlation analyses were performed between three spirometry parameters: forced expiratory volume in 1 s, forced vital capacity, and diffusing capacity of the lung for carbon monoxide; and four main capillaroscopy alterations: presence of neoangiogenesis, pericapillary stippling, low capillary density, and inverted capillary apex, as listed in the tables below. There was no significant difference in PFTs between the presence and absence of low capillary density, neoangiogenesis, pericapillary stippling, and inverted capillary apex in EGPA patients (Table 5, Table 6, Table 7 and Table 8).

Overall, these results indicate that no significant correlation was found between all these capillaroscopy alterations and PFTs.

### 3.3. Influence of C-Reactive Protein Levels on Capillaroscopy Alterations in EGPA Patients

We collected the C-reactive protein levels of EGPA patients obtained within approximately ±6 months of the capillaroscopy examination. To evaluate the potential utility of capillaroscopy as a marker of active disease status, we compared the four main capillaroscopic alterations with C-reactive protein levels, which we considered an indicator of active inflammatory disease; the results are listed below. There was no significant difference in C-reactive protein levels presence and absence of low capillary density, neoangiogenesis, pericapillary stippling and inverted capillary apex in EGPA patients (Table 9, Table 10, Table 11 and Table 12).

Overall, these results indicate that no significant correlation was found between all these capillaroscopy alterations and C-reactive protein levels.

### 3.4. Differences in Capillaroscopy Alterations Between pANCA-Negative and pANCA-Positive EGPA Patients

In line with the current scientific literature, we aimed to assess whether statistically significant differences existed between p-ANCA-positive and p-ANCA-negative patients within the EGPA cohort. Specifically, we evaluated potential statistical differences in the four capillaroscopic abnormalities under investigation. In addition, we compared the two ANCA subgroups (p-ANCA-positive vs. p-ANCA-negative) with respect to C-reactive protein levels and PFTs; the results are listed below (Table 13, Table 14 and Table 15).

No statistically significant differences were identified between the two groups with regard to the four capillaroscopic abnormalities analyzed.

There was no significant difference in PFTs between the two ANCA subgroups (p-ANCA-positive vs. p-ANCA-negative).

There was no significant difference in C-reactive protein levels between the two ANCA subgroups (p-ANCA-positive vs. p-ANCA-negative). These results suggest that no statistically significant differences emerged between the two EGPA subgroups for the parameters assessed.

## 4. Discussion

In this study, we aimed to analyze nailfold videocapillaroscopy to assess its potential role in EGPA patients. Firstly, we identified four major NVC abnormalities that showed statistically significant differences compared to healthy controls. Secondly, the association between capillaroscopy alterations and C-reactive protein levels did not reach statistical significance, which may be attributed to the limited sample size of the EGPA cohort, potentially underpowering the analysis. Third, the age- and sex-adjusted percent predicted values of the three evaluated spirometry parameters demonstrated no statistically significant correlation with the observed capillaroscopic abnormalities. Finally, we potentially identified a capillaroscopic EGPA pattern, defined by the presence of these four specific NVC alterations, which may offer valuable diagnostic support by enabling the inclusion of a larger number of individuals in the disease suspicion framework, potentially facilitating earlier identification and recruitment of patients in the initial stages of the disease. However, this approach inevitably raises the concern of large-scale screening leading to a substantial rate of false positives. Therefore, it is essential to further investigate these two key alterations in larger cohorts and in comparison with other diseases to better establish their specificity and diagnostic utility. Only a limited number of studies in the literature have aimed to characterize nailfold videocapillaroscopy findings in vasculitis. In 2022, Keret et al. investigated this phenomenon in 25 patients, 7 of whom were diagnosed with ANCA-associated vasculitis, including 2 with EGPA, comparing them to a matched healthy control group [31]. Matsuda et al. analyzed capillaroscopic alterations in 51 patients with ANCA associated vasculitis, among whom 9 had EGPA [27]. Rimar et al. evaluated 17 patients with active vasculitis, including 3 with EGPA [32]. All the cited studies demonstrate statistically significant NVC findings in ANCA-associated vasculitis, despite including only a small number of patients with EGPA, highlighting the limited representation of this subgroup in the current literature. Our study shows in a larger sample that in patients with EGPA, capillaroscopic neoangiogenesis, inverted capillary apex, rolling, and pericapillary stippling occur in statistically significant manners in comparison with healthy subjects. These results corroborate the observations reported in previous studies, expanding the evidence base by analyzing a larger patient cohort. The findings of our study confirm the presence of microcirculatory alterations in patients with EGPA. However, none of our patients showed NVC-specific abnormalities (such as giant capillaries or avascular areas), as are frequently observed in scleroderma spectrum disorders. Moreover, the microcirculation alterations were also compared to patients’ spirometry values with no correlation of reduced density, rolling, pericapillary stippling, inverted capillary apex, and presence of neoangiogenesis with FVC, FEV1, and DLCO. The analysis of the association between the five capillaroscopic alterations and C-reactive protein levels resulted in *p*-values above the conventional significance of 0.05, indicating a lack of statistical significance. However, this conclusion is not definitive, as there was a noticeable trend toward significance with *p*-values approaching 0.05. This limitation is primarily attributable to the absence of a standardized approach for utilizing CRP as a biomarker of disease activity in this setting. Owing to the absence of data at the time of patient enrolment and/or an excessive time interval between capillaroscopy and clinical assessment and laboratory tests, which would have otherwise compromised the validity of the observed correlation, we had to assess disease activity using CRP levels, without utilizing the standardized BVAS v3 score [33]. Finally, Emmi et al. previously described the differences observed between ANCA-positive and ANCA-negative EGPA populations, noting ANCA positivity in approximately 30–40% of EGPA cases [5]. Starting from this evidence, we investigated whether similar distinctions could be observed in capillaroscopic patterns, pulmonary function tests, and C-reactive protein levels. In our cohort, no statistically significant differences emerged across these parameters. However, based on clinical experience and observations from the patients enrolled in this study, differences between the two subgroups, particularly in terms of organ involvement, appear to persist. Such findings may not have emerged due to the limitations of our study design. Therefore, further studies are needed to better characterize EGPA subtypes and, eventually, any pathogenetic differences between ANCA-positive and ANCA-negative in EGPA patients, potentially incorporating additional biomarkers and by evaluating any differences among the NVC abnormalities.

### 4.1. Subanalysis of Patients with EGPA and Healthy Controls, Role of NVC

The presence of capillary alterations in AAV is a finding that already exists in the literature. Considering the studies that have investigated this aspect, capillaroscopy alterations have been identified in approximately 70–80% of patients with AAV, distinguishing them from the various control groups. However, in many case series, these alterations were described as non-specific or minor (e.g., increased tortuosity, microhemorrhages, and enlarged, bushy, and bizarre capillaries, and with recurrent architectonic disarrangement). A 2023 study comparing capillaroscopic alterations in patients with ANCA-associated vasculitis and healthy controls found statistically significant differences, including microhemorrhages, neoangiogenesis, capillary loss, and increased tortuosity in the AAV group [27]. Furthermore, the authors proposed a potential correlation between these capillaroscopic abnormalities and the severity of organ involvement in AAV, and evidence showed that, after three months of therapy, the number of microhemorrhages was significantly reduced. On the other hand, two studies that compared capillaroscopic alterations in AAV patients with a control group reported no statistically significant differences between the two populations regarding these findings [34,35,36]. However, certain considerations should be made regarding these studies. Trigiannese et al. included only AAV patients without comorbidities in the disease group, which may partially explain the lack of significant capillaroscopy abnormalities compared to healthy controls [34]. Similarly, Sullivan et al. selected only AAV patients in a documented state of remission, all of whom were undergoing long-term therapy with the disease under complete control [35]. This may have attenuated the inflammatory activity, thus accounting for the absence of significant differences with the control group. However, it is important to report a similar limitation in the study by Matsuda et al., who included only patients with advanced-stage disease, potentially associated with a highly progressed state of vascular damage [27]. However, this consideration may help explain the underlying reasons for the identification of capillaroscopy alterations in patients with AAV. For example, considering neoangiogenesis as an isolated capillaroscopic finding, which is the physiological or pathological process of new capillary blood vessel sprouting and growth from existing vasculature, it represents an important marker of vascular inflammatory activity, highlighting this mechanism of injury as a predominant feature in AAV. In fact, in our study, which did not take disease activity status into account, reflecting a more heterogeneous patient population, neoangiogenesis was particularly prominent, with a percentage of 72%, and was greater when compared to the health control group. In contrast, the reduction in capillary density, which can likewise be interpreted as a marker of microvascular damage, was more frequently observed in the EGPA group, with a prevalence of 38% [34,35,36]. However, when compared to the control group, in which it was present in 21% of cases, this difference did not reach statistical significance. This finding, given the *p*-value’s tendency toward 0.05, may be attributed to the limited sample size of the EGPA group, which may have prevented the result from achieving statistical significance. This highlights one of the main limitations in the assessment of rare diseases, which is the small study population. A 2022 study that aimed to assess the possible role of NVC in systemic vasculitis has introduced a novel capillaroscopy alteration not previously evaluated, which is pericapillary stippling, and it was markedly present in patients with active disease [31]. This finding may represent a capillaroscopy marker indicative of disease activity. In our study, pericapillary stippling was particularly prominent within the EGPA population with a percentage of 66% and was greater when compared to the healthy control group. This alteration likely reflects the extravasation of red blood cells from structurally compromised capillaries, leading to pericapillary iron deposition that subsequently undergoes conversion into hemosiderin. Similarly, this study also described the presence of rolling as being markedly more prevalent in the patient population with active disease [31]. In our study as well, rolling was particularly prominent with a percentage of 100% and was significantly greater compared to the healthy control group. This finding requires further evaluation to understand how all patients with EGPA, not only those in the active phase, exhibited this capillaroscopic alteration. Specifically, in our study, although the BVAS v3 score was not preliminarily assessed, three patients were not receiving any treatment as they had achieved a state of well-being and disease control that did not require therapy. Despite this evident absence of active disease, these patients nonetheless exhibited the same alteration. In actuality, we did not identify a specific capillaroscopic pattern that can be used for the early diagnosis of EGPA, or any pattern capable of assessing disease activity. However, it is evident that the presence of these four capillaroscopic alterations should always be evaluated in patients with a suspected diagnosis or those with an early stage of this disease, such as refractory asthma or persistent hypereosinophilia, as these should be considered as potential red flags. Their identification can facilitate earlier diagnosis and can enable early treatment initiation. This approach may help prevent adverse disease progression involving multiple organs, which can lead to poor prognosis or reduced quality of life.

### 4.2. The Primary Limitations Encountered Throughout the Study

The assessment of capillaroscopy utilization in EGPA patients has been influenced by various factors, including the following:

- A key limitation of this study is the small sample size, which is attributable to the rarity of the disease under investigation. While no definitive conclusions can be drawn, the trend of several *p*-values approaching 0.05 suggests that a larger cohort might have clarified whether certain observed differences reach statistical significance or confirmed the absence of significant associations.
- An additional limitation of this study is the heterogeneity of treatment regimens among participants. Moreover, while most of the patients were receiving corticosteroids or biologic therapies, three patients were not receiving any specific treatment due to stable disease control. Owing to the rarity of the condition and the limited sample size, it was not possible to perform an analysis based on treatment regimen.
- Furthermore, another limitation of the study is the extended recruitment period, ranging from October 2023 to April 2025.

## 5. Conclusions

Our study confirmed the presence of four microcirculatory abnormalities in patients with EGPA, in particular with regard to pericapillary stippling, rolling, inverted capillary apex, and neoangiogenesis. Currently, these abnormalities do not appear to be associated with any specific clinical or pathological feature of the disease. Although the data are derived from a limited cohort, nevertheless, our study represents the largest sample reported in the literature to date, and is the only study that has focused exclusively on EGPA. The most compelling findings, which need further investigation in larger cohorts and over extended follow-up periods, concern the use of the four capillaroscopic alterations cited as potential red flags for the early diagnosis of EGPA. Another aspect that requires further investigation is the potential use of capillaroscopy in the follow-up of these patients, with the aim of predicting disease relapses by assessing the state of microvascular inflammation. Overall, given the current lack of validated markers in EGPA capable of providing an early diagnostic suspicion and disease progression, capillaroscopy, being a non-invasive and easily performed technique, should be considered in these patients to better understand its true clinical utility.

## Figures and Tables

**Table 1:** Normal capillaroscopy parameters.

Capillary array, architecture, morphology	Homogeneous distribution of U-shaped capillaries, perpendicular to the nailfold.
Capillary tortuosity	Typically absent.
Dilated and/or giant capillaries	Not present.
Ramified capillaries	Not present.
Neoangiogenesis	Not present.
Hemorrhages, hemosiderin deposits	Typically not present, may be show after local trauma.
Capillary number	Normal capillary density is about 9 to 14 capillaries per mm.
Avascular areas	Not present.
Capillary blood flow	Dynamic, without stasis of red blood cells.

**Table 2:** Characteristics of our study population.

Characteristic	N = 29 ^1^
Sex	
F	19 (66%)
M	10 (34%)
Age	62 (51, 72)
Age at diagnosis	58 (50, 73)
Unknown	9
Smokers	20 (69%)
Organ involvement	
Lung	29 (100%)
ENT	27 (93%)
Skin	3 (10%)
GI	8 (28%)
Heart	7 (24%)
Joint	4 (14%)
Neural	5 (17%)
Other	5 (17%)
Comorbidities	
Obstructive Sleep apnea (OSA)	1 (3%)
Chronic kidney disease (CKD)	(7%)
PFR data	
FEV1, as % predicted	86 (81, 98)
FVC, as % predicted	101 (94, 113)
DLCO, as % predicted	85 (78, 93)
Unknown	3
HRTC patterns	
GGO	5 (17%)
Consolidations	12 (41%)
Nodules	10 (34%)
Bronchiectasis	3 (10%)
CRP mg/L	1.50 (1.10, 2.50)
ANCA+	10 (34%)

^1^ n (%); median (Q1, Q3). Legend: F = female, M = male, ENT = ear, nose, and throat, GI = gastrointestinal, PFR = pulmonary function tests, FEV1 = forced expiratory volume in one second; FVC = forced vital capacity, DLCO = diffusing capacity of the lung for carbon monoxide, GGO = ground glass opacity, CRP = C-reactive protein, ANCA = antineutrophil cytoplasmic antibodies.

**Table 3:** Treatment of our study population.

Ongoing Therapy	N = 29 ^1^
Steroid	15 (52%)
Methotrexate	6 (21%)
Mycophenolate	1 (3%)
Azathioprine	2 (7%)
Mepolizumab	11 (38%)
Benralizumab	5 (17%)
Dupilumab	1 (3%)

^1^ n (%).

**Table 4:** Microcirculation abnormalities and statistical analysis between healthy population and EGPA.

Status	Case N = 29 ^1^	Control N = 29 ^1^	*p*-Value ^2^
Sex			>0.9
F	19 (66%)	19 (66%)	
M	10 (34%)	10 (34%)	
Age	62 (51, 72)	58 (52, 70)	0.4
Smokers	20 (69%)	19 (66%)	0.3
Low capillary density	11 (38%)	6 (21%)	0.15
Neoangiogenesis	21 (72%)	2 (6.9%)	<0.001
Pericapillary stippling	19 (66%)	2 (6.9%)	<0.001
Rolling	29 (100%)	5 (17%)	<0.001
Inverted capillary apex	15 (52%)	3 (10%)	<0.001

^1^ n (%); median (Q1, Q3); ^2^ Pearson’s chi-squared test; Wilcoxon rank sum test.

**Table 5:** Statistical analysis between low capillary density and PFTs.

Low Capillary Density	Absent N = 18 ^1^	Present N = 11 ^1^	*p*-Value ^2^
FEV1, as % predicted	88 (23)	89 (16)	>0.9
FVC, as % predicted	105 (20)	103 (17)	0.8
DLCO, as % predicted	83 (17)	84 (16)	>0.9
Unknown	2	1	

^1^ Mean (SD); ^2^ Welch two-sample *t*-test.

**Table 6:** Statistical analysis between neoangiogenesis and PFTs.

Neoangiogenesis	Absent N = 8 ^1^	Present N = 21 ^1^	*p*-Value ^2^
FEV1, as % predicted	89 (32)	88 (16)	>0.9
FVC, as % predicted	107 (27)	103 (15)	0.7
DLCO, as % predicted	86 (22)	82 (13)	0.7
Unknown	0	3	

^1^ Mean (SD); ^2^ Welch two-sample *t*-test.

**Table 7:** Statistical analysis between pericapillary stippling and PFTs.

Pericapillary Stippling	Absent N = 10 ^1^	Present N = 19 ^1^	*p*-Value ^2^
FEV1, as % predicted	87 (20)	89 (22)	0.8
FVC, as % predicted	104 (11)	104 (22)	0.9
DLCO, as % predicted	85 (14)	83 (17)	0.8
Unknown	2	1	

^1^ Mean (SD); ^2^ Welch two-sample *t*-test.

**Table 8:** Statistical analysis between inverted capillary apex and PFTs.

Inverted Capillary Apex	Absent N = 14 ^1^	Present N = 15 ^1^	*p*-Value ^2^
FEV1, as % predicted	93 (18)	84 (23)	0.2
FVC, as % predicted	106 (18)	103 (19)	0.7
DLCO, as % predicted	87 (16)	80 (16)	0.3
Unknown	2	1	

^1^ Mean (SD); ^2^ Welch two-sample *t*-test.

**Table 9:** Statistical analysis between low capillary density and CRP.

Low Capillary Density	Absent N = 18 ^1^	Present N = 11 ^1^	*p*-Value ^2^
CRP mg/L	2.15 (0.70, 3.60)	1.34 (1.10, 2.10)	0.5

^1^ Median (Q1, Q3); ^2^ Wilcoxon rank sum test.

**Table 10:** Statistical analysis between neoangiogenesis and CRP.

Neoangiogenesis	Absent N = 8 ^1^	Present N = 21 ^1^	*p*-Value ^2^
CRP mg/L	1.20 (0.85, 1.95)	2.10 (1.10, 3.60)	0.4

^1^ Median (Q1, Q3); ^2^ Wilcoxon rank sum test.

**Table 11:** Statistical analysis between pericapillary stippling and CRP.

Pericapillary Stippling	Absent N = 10 ^1^	Present N = 19 ^1^	*p*-Value ^2^
CRP mg/L	1.90 (1.20, 3.60)	1.34 (0.60, 2.50)	0.4

^1^ Median (Q1, Q3); ^2^ Wilcoxon rank sum test.

**Table 12:** Statistical analysis between inverted capillary apex and CRP.

Inverted Capillary Apex	Absent N = 14 ^1^	Present N = 15 ^1^	*p*-Value ^2^
CRP mg/L	1.15 (0.50, 3.60)	2.10 (1.20, 2.50)	0.2

^1^ Median (Q1, Q3); ^2^ Wilcoxon rank sum test.

**Table 13:** Statistical analysis between capillaroscopy alterations and the two subgroups (p-ANCA-positive and p-ANCA-negative).

ANCA+	Absent N = 19 ^1^	Present N = 10 ^1^	*p*-Value ^2^
Low capillary density	7/19 (37%)	4/10 (40%)	>0.9
Neoangiogenesis	14/19 (74%)	7/10 (70%)	>0.9
Rolling			
1	19/19 (100%)	10/10 (100%)	
Pericapillary stippling	13/19 (68%)	6/10 (60%)	0.7

^1^ n/N (%); ^2^ Fisher’s exact test.

**Table 14:** Statistical analysis PFTs and the two subgroups (p-ANCA-positive and p-ANCA-negative).

ANCA+	Absent N = 19 ^1^	Present N = 10 ^1^	*p*-Value ^2^
FEV1, as % predicted	86 (21)	94 (21)	0.3
FVC, as % predicted	103 (19)	107 (19)	0.5
DLCO, as % predicted	84 (17)	82 (16)	0.7
Unknown	3	0	

^1^ Mean (SD); ^2^ Welch two-sample *t*-test.

**Table 15:** Statistical analysis CRP and the two subgroups (p-ANCA-positive and p-ANCA-negative).

Eval	Absent N = 19 ^1^	Present N = 10 ^1^	*p*-Value ^2^
CRP mg/L	1.34 (0.60, 3.80)	1.60 (1.20, 2.22)	>0.9

^1^ Median (Q1, Q3); ^2^ Wilcoxon rank sum test.

## Data Availability

All the data are available upon reasonable request to the corresponding author.

## Abbreviations

The following abbreviations are used in this manuscript:

AAV	Antineutrophil cytoplasmic antibody-associated vasculitis
EGPA	Eosinophilic granulomatosis with polyangiitis
MPA	Microscopic polyangiitis
GPA	Granulomatosis with polyangiitis
p-ANCA	Perinuclear antineutrophil cytoplasmic antibodies
MPO	Myeloperoxidase
PR3	Proteinase 3
ANA	Antinuclear antibodies
ENA	Extractable nuclear antigen
ANCA	Antineutrophil cytoplasmic antibodies
IgE	Immunoglobulin E
NVC	Nailfold videocapillaroscopy
PFTs	Pulmonary function tests
HRTC	High-resolution computed tomography
CRP	C-Reactive Protein
FVC	Forced vital capacity
FEV1	Forced expiratory volume in 1 second
DLCO	Diffusing capacity of the lung for carbon monoxide
FFS	Five factor score
ACR	American College of Rheumatology
BVAS	Birmingham Vasculitis Activity Score
RP	Raynaud’s phenomenon
SSc	Systemic sclerosis
